# Supplementary material for: The Evolution and Characterization of the RNA Interference Pathways in Lophotrochozoa
Source: Genome Biol Evol. 2024 May 7;16(5):evae098. doi: 10.1093/gbe/evae098 (PMC11114477; doi:10.1093/gbe/evae098)
Supplement: evae098_Supplementary_Data [file evae098_supplementary_data.zip › Supplementary_materials.zip!/selected_miRNAs/NoGe_selected_miRNAs/known_miRNA_families_pdf_files/NMRB01000117.1_Notospermus_geniculatus_strain_Ushimado_scaffold117,_whole_genome_shotgun_sequence_4718.pdf]

Provisional ID	:	NMRB01000117.1_Notospermus_geniculatus_strain_Ushimado_scaffold117,_whole_genome_shotgun_sequence_4718
Score total	:	25499.6
Score for star read(s)	:	3.9
Score for read counts	:	25491.4
Score for mfe	:	1.3
Score for randfold	:	0
Score for cons. seed	:	3
Total read count	:	50012
Mature read count	:	49980
Loop read count	:	0
Star read count	:	32

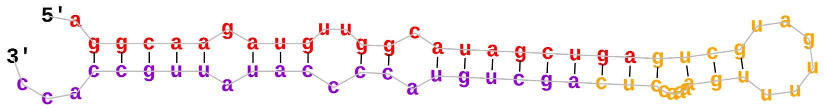

Genome coordinates: NMRB01000117.1 413527-413592

pre-miRNA sequence:
AGGCAAGAUGUUGGCAUAGCUGAAGGCAAGAUGUUGGCAUAGCUGAGUCGUAGUUUUGAAAACCUCAGCUGUACCCCAUAUUGCCACC

Mature sequence: AGGCAAGAUGUUGGCAUAGCUGA

Aligned mature sequence of the *N. geniculatus* pre-miRNA with the top 5 hits from the BLAST search against the pre-miRNA mirGeneDB set:

```
                                     **********************
    Nge_Mir-31                       aggcaagauguuggcauagcuga
    Sko-Mir-31_5p                    aggcaagauguuggcauagcugu
    Lan-Mir-31-P6_5p                 aggcaagauguuggcauagcuga
    Lan-Mir-31-P7_5p                 aggcaagauguuggcauagcuga
    Npo-Mir-31_5p                    aggcaagauguuggcauagcuga
    Lgi-Mir-31_5p                    aggcaagauguuggcauagcuga
```
